# Transcriptomic Signature of PDGF-BB Control of Annulus Fibrosus Reveals Modulation of Inflammatory and Neurogenic Pathways

**DOI:** 10.3390/cells15111007

**Published:** 2026-05-30

**Authors:** Changli Zhang, Gilbert Gu, Joshua W. McNulty, David Berenfeld, Lisbet Haglund, Sangwook Tim Yoon, Brian Goh, Hicham Drissi

**Affiliations:** 1 Department of Orthopaedics, School of Medicine, Emory University, Atlanta, GA 30329, USA; gilbert.gu@emory.edu (G.G.); jmcnulty8@gatech.edu (J.W.M.); david.berenfeld@emory.edu (D.B.);; 2 Department of Surgery, McGill University, Montreal, QC H4A3J1, Canada; 3 Emory Orthopaedics & Spine Center, Atlanta, GA 30329, USA; 4 Atlanta Veterans Affairs Medical Center, Decatur, GA 30033, USA

**Keywords:** PDGF, annulus fibrosus, inflammation, neurogenesis, intervertebral disc degeneration

## Abstract

**Highlights:**

**What are the main findings?**

- PDGF-BB treatment alters the transcriptional levels of genes associated with cell cycle progression, extracellular matrix remodeling, inflamation, and neurogenic singlaing in human annulus fibrosus cells.
- TNF-α induced the expression of inflammatory and pain-related mediators, including nerve growth factos, endothelin 1, and interleukin 6 and 8, while PDGF-BB inhibited these responses in human annulus fibrosus cells.

**What are the implications of the main findings?**

- PDGF-BB may improve annulus fibrosus cell function under inflammatory conditions associated with intervetebral disc degeneration.
- These findings provide a transcriptomic foundation for further investigation of the impact of PDGF-BB in disc inflammation and pain-related signaling.

**Abstract:**

Low back pain is closely associated with intervertebral disc (IVD) degeneration, in which inflammation and neovascularization within the annulus fibrosus (AF) contribute to pain generation. Platelet-derived growth factor (PDGF)-BB plays a crucial role in tissue repair and cellular homeostasis, but its role in AF cell biology remains poorly understood. To investigate the effects of PDGF-BB on human AF cells, healthy and degenerated AF cells were treated with PDGF-BB for 3 or 5 days, followed by bulk RNA sequencing. Functional enrichment of differentially expressed genes, transcription factor activity analysis, and protein–protein interaction network analysis was performed. Publicly available single-cell RNA-seq data were used to compare the transcriptomic profiles of native healthy and degenerated AF samples. In addition, TNF-α stimulation was conducted to validate the anti-inflammatory effects of PDGF-BB. Our findings suggest that PDGF-BB induced both common and context-dependent transcriptional responses in healthy and degenerated AF cells. In healthy AF cells, PDGF-BB consistently upregulated genes associated with cell cycle and developmental growth. In degenerated AF cells, PDGF-BB also induced these responses, while additionally it downregulated the genes related to extracellular matrix remodeling and collagen degradation. Meanwhile, PDGF-BB showed common effects in both healthy and degenerated cells by modulating the expression of genes within G protein-coupled receptor (GPCR) networks that are linked to complement, inflammation, and neurotransmitter signaling. In addition, PDGF-BB also suppressed the expression of genes involved in inflammatory-neurogenic signaling, including nerve growth factor (*NGF*), C-X-C motif chemokine ligand 12 (*CXCL12*), and apolipoprotein E (*APOE*). To relate these PDGF-BB induced responses to disc degeneration, we reanalyzed publicly available single-cell RNA-seq datasets from native human AF tissues and found that *NGF*-positive cells showed increased tumor necrosis factor (TNF)-α signaling activity. When AF cells were stimulated with TNF-α, PDGF-BB treatment significantly inhibited the expression of *NGF*, endothelin-1 (*EDN1*), and interleukin 6 (*IL6*) under both baseline and TNF-α-stimulated conditions. These results suggest that PDGF-BB modulates gene expression associated with inflammatory and neurogenic signaling as well as ECM remodeling in human AF cells, providing a transcriptomic insight into the PDGF-BB’s function in AF biology.

## 1. Introduction

Low back pain (LBP), a leading cause of years lived with disability, represents a significant global health burden on both an individual and economic scale [1,2]. It is estimated that LBP costs $100–200 billion annually in the United States alone [3]. The staggering prevalence and cost of LBP necessitates research into understanding the underlying causes and pathology. It has been reported that approximately 40% of LBP cases are associated with intervertebral disc (IVD) degeneration [4,5], suggesting its central role in the pathogenesis of LBP.

The IVD consists of three unique compartments: the nucleus pulposus (NP), annulus fibrosus (AF), and the cartilaginous end plate (EP) [6,7]. The AF consists of collagen layers that join the vertebra together, allows interbertebral motion, and contains the NP which consists of a visco-elastic proteoglycan/collagen matrix within the center of the disc [8,9]. Degeneration of the AF region is marked by physical features such as radial fissures and disorganization of regular lamellar patterns [8,10]. This degeneration can subsequently lead to decreased disc height and a reduced load bearing capacity.

With aging and IVD degeneration, inflammation progressively accumulates within the AF, contributing to structural deterioration and the upregulation of pain-related mediators, which contribute to heightened pain sensation and subsequent low back pain [11,12]. Notably, systemic overexpression of human TNF-α, a key pro-inflammatory cytokine, induced pronounced degenerative changes in the AF of mouse caudal IVDs, characterized by cell death, altered collagen matrix, and infiltration of immune cells in mouse caudal IVDs, while exerting minor effects on the NP [13]. In vitro, TNF-α stimulation in both AF and NP cells further induced an upregulation of inflammatory and neurogenic mediators including *IL6*, *NGF*, and *EDN1*, highlighting the critical role of inflammatory signaling in disc degeneration and pain generation [14,15].

Platelet-derived growth factor (PDGF) BB is a major component of platelet-rich plasma (PRP) that has been widely studied in preclinical models and clinical settings for reducing pain and promoting tissue regeneration and repair [16,17]. PDGF-BB activates receptor-mediated cascades of phosphorylation that regulate cell proliferation, migration, and survival pathways [18]. We have previously established recombinant human (rh) PDGF-BB as an inhibitor of human NP cell apoptosis with an anabolic effect on matrix production in human degenerated NP cells isolated from Pfirrmann grade II or III NP tissues [19]. Furthermore, when delivered via hydrogel, rh PDGF-BB successfully inhibited the progression of puncture-induced IVD degeneration and restored biomechanical function in a rabbit preclinical model [20]. In our recent work, we demonstrated that PDGF-AB and -BB reduced disc degeneration through suppressing the senescent phenotype. We found that in NP cells, treatment with PDGF-AB and -BB upregulated the expression of PDGF receptor α (*PDGFRA*) and cell cycle-related genes, while downregulating the expression of genes associated with reactive oxygen species (ROS) production, oxidative stress, and mitochondrial dysfunction [21]. By contrast, in AF cells, PDGF treatment reduced the senescent phenotype without significantly affecting the gene expression levels of PDGFRA [21].

Despite this progress, the effects of PDGF-BB on AF cells, particularly the differential responses between healthy and degenerated AF cells, remain poorly understood. In this study, we aimed to investigate changes in transcriptome profiles in healthy and degenerated human AF cells in response to PDGF-BB treatment and examine the effects of PDGF-BB on inflammatory and neurotrophic signaling under both basal and TNF-α-induced conditions.

## 2. Materials and Methods

### 2.1. Isolation of AF Cells

All experimental procedures were approved by the institutional review board of Emory University (IRB #00099028, approval date 28 February 2018). After written consent was obtained, human severely degenerated AF tissues (Grade IV or V on Pfirrmann grade) were obtained after discectomy surgeries for discogenic pain. Cells were isolated from the tissues as described before [21] and cultured in low-glucose DMEM, containing 10% fetal bovine serum (FBS) (VWR, Radnor, PA, USA, 189870654), 1× antibiotic-antimycotic (Thermofisher Scientific, Waltham, MA, USA, 15240062), and 50 µg/mL L-Ascorbic acid 2-phosphate (Sigma, St. Louis, MO, USA, A8960). Cells were grown at 37 °C under 5% CO_2_ and 20% O_2,_ and passage 3 cells were used for each experiment. Healthy AF cells were gifted by Dr. Lisbet Haglund from McGill University. The donor information is provided in Table 1. The mean ages for healthy donors are 22.4 ± 3.44 years and for degenerated donors are 64.67 ± 9.33 years.

### 2.2. PDGF Treatment in Human Annulus Fibrosus Cells

Human degenerated (*n* = 6) and healthy (*n* = 5) AF cells were seeded in 6-well plates (2 × 10^4^ cells/mL), followed by serum deprivation in 0.2% FBS media for 1 day. Cells were then treated with recombinant human (rh) PDGF-BB (20 ng/mL; PeproTech, Cranbury, NJ, USA, 10771918) for 3 or 5 days. For TNF-α stimulation experiments, healthy and degenerated AF cells (n = 4 for each group) were serum-deprived in DMEM containing 0.2% FBS for 1 day, and then assigned into three groups: (1) control (0.2% FBS only), (2) TNF-α (20 ng/mL; Biotechne, Minneapolis, MN, USA, 210-TA020), (3) TNF-α (20 ng/mL) + PDGF-BB (20 ng/mL) for an additional 1 or 3 days to evaluate the effects of PDGF-BB on TNF-α-stimulated cells. Treatments were maintained for 1 or 3 days, with culture media changes every other day. The concentration and treatment duration of TNF-α was selected based on the previous literature investigating the NGF expression in TNF-α stimulated human AF cells [22].

### 2.3. RNA Sequencing

For bulk RNA sequencing, total RNA was isolated with TRIzol (Thermofisher Scientific, 15596018) and purified using the miRNeasy kit (Qiagen, Germantown, MD, USA, 217084). RNA concentration and integrity number were determined using Agilent TapeStation 4150 (Agilent Technologies, Santa Clara, CA, USA, G2992AA). RNA samples (~500 mg) with RIN > 7 were shipped to Novogene (Novogene, Sacramento, CA, USA) for library construction and sequencing. Pair-end RNA sequencing was performed on the Illumina Novaseq platform (Novogene, Sacramento, CA, USA) with an average of 20 million reads per sample as previously described [21]. Low-quality reads and adapters were removed after sequencing. The quality of raw sequencing reads was further assessed by FastQC (fastqv_v0.12.1) before downstream analysis, including per base sequence quality and content, per sequence quality scores and GC contents, sequence length distribution, and sequence duplication levels. The sequencing reads were then mapped to the human reference genome sequence using DNASTAR Navigator 17, and raw read counts were extracted after alignment. Overall transcriptomic differences between untreated and treated samples were visualized by distinct clustering from principal component analysis (PCA), which was performed based on the top 500 most variable genes in each sample. These multidimensions were projected onto the first two principal components and displayed in PCA plots using R (version 4.4.3). The raw sequencing files were uploaded into NCBI Sequence Read Archive (SRA) database (PRJNA1354819). In addition, previously published RNA seq data from degenerated AF cells treated with PDGF-BB for 5 days (PRJNA1150962) were reanalyzed and integrated in this study [21]. In our previous work, we analyzed degenerated NP and AF cells treated with PDGF-AB or PDGF-BB for 5 days. In the present study, we extend this work by focusing on both healthy and degenerated AF cells and incorporating a time-course analysis at 3 and 5 days following PDGF-BB treatment. Bulk RNA sequencing was performed in the same batch for the samples used in both previous and current studies.

### 2.4. Differential Gene Expression Analysis of RNA-Seq Data

To get the differentially expressed genes (DEGs), pairwise gene expression comparison was performed between untreated and PDGF-BB-treated groups at each timepoint in healthy or degenerated conditions. DEGs were identified by using DESeq2 (version 1.46.0) package in R (version 4.4.3), which took raw read counts extracted from DNASTAR alignment as an input. Genes with less than total 10 counts across all samples were filtered out before downstream analysis, and genes were considered as DEGs with absolute fold change no less than 2 and adjusted *p* value less than 0.05. DEGs were visualized by heatmap and volcano plots. Heatmap plots were generated using pheatmap package (version 1.0.13) and volcano plots were generated using ggplot2 package (version 4.0.0) in R (version 4.4.3).

### 2.5. Pathway Enrichment Analysis

Gene ontology (GO) analysis was performed using upregulated and downregulated DEGs separately in R (version 4.4.3) and visualized by clustered heatmap analysis of DEGs with functional enrichment annotation in Cytoscape (version 3.10.3). Functional enrichment network plots were generated to show the largest network which was clustered based on functional similarity. Over-Representation Analysis (ORA) of both upregulated and downregulated DEGs was conducted using the Reactome pathway database to identify significantly enriched biological pathways in R (version 4.4.3). Gene Set Enrichment Analysis (GSEA) was performed using the Molecular Signatures Database (MSigDB) Hallmark gene sets to identify the pathway shifts induced by PDGF-BB treatment between healthy and degenerated AF cells at each timepoint.

### 2.6. Transcription Factor Activity Inference

To determine the upstream transcriptional regulators of PDGF-BB treatment, transcription factor activity was inferred from bulk RNA sequencing using human DoRothEA regulons in R (version 4.4.3). Normalized DEGs shared between day 3 and day 5 timepoints in each condition were used as the input gene set. The activity of the transcription factor was calculated based on the enrichment of the target genes within each regulon. The activity score of each transcription factor reflects the predicted activation, with a higher score indicating increased activity and a lower score indicating decreased activity. The activity score was then compared between untreated and PDGF-BB-treated samples in both healthy and degenerated AF cells.

### 2.7. Protein–Protein Interaction (PPI) Network Analysis

The PPI network was constructed from shared DEGs by PDGF-BB treatment in both healthy and degenerated AF cells at day 3 and day 5. Interactions were retrieved from the STRING database and visualized in Cytoscape (version 3.10.3). Hub genes were identified using the cytoHubba plugin. Nodes were ranked based on Maximal Clique Centrality (MCC) and the top 10 hub genes were displayed to show the interaction of key genes regulated by PDGF-BB.

### 2.8. Single Cell RNA Sequencing Analysis

To compare transcriptomic differences between healthy and degenerated human AF cells from native tissues, we re-analyzed the publicly available single-cell RNA-seq datasets obtained from GEO databases, including health AF samples from GSE229711 and degenerated AF samples from GSE230808 [23]. Cells expressing less than 200 or more than 7000 unique genes were excluded as low-quality cells and potential doublets. To further reduce the influence of doublets, cells in the top 3% of total counts were filtered out. Additionally, cells with mitochondrial genes exceeding 5% of the total gene counts were considered as low-quality cells and removed. After initial QC, samples with a cell number less than 1000 were excluded. In total, three healthy samples with a total of 20,450 cells and six degenerated samples (Thompson grade 3) with 17,360 cells were used for analysis. Data were then normalized and integrated using the Harmony algorithm to reduce the inter-donor variability in the Seurat R package (version 5.3.1). A resolution of 0.5 was used with uniform manifold approximation and projection (UMAP) visualization. To annotate each cell cluster, differentially expressed marker genes of each cluster were identified by the FindMarkers function in Seurat with Wilcoxon rank-sum test and used for cluster annotation. For each cluster, cells were compared against cells in all the other clusters to identify genes enriched in that cluster. To investigate role of nerve growth factor (*NGF*) in AF cells, cells were classified into *NGF*-positive and *NGF*-negative groups based on *NGF* expression levels. Cells with no detectable *NGF* transcript were considered *NGF*-negative population, whereas cells with *NGF* gene counts greater than 0 were considered *NGF*-positive population. To investigate the pathways associated with *NGF*-positive and *NGF*-negative cell populations, co-expressed genes within each population were identified using correlation-based analysis within each population. Pathway enrichment analysis was subsequently performed on these genes using clusterProfiler with hallmark gene sets from MSigDB (Molecular Signature Database) in R (version 4.4.3). Hallmark gene sets with a false discovery rate (FDR) less than 0.05 were considered significant. 

After identifying that *NGF*-positive cells were significantly associated with the pathway of TNF-α signaling via NF-kB, we further investigated which AF cell populations were enriched in this pathway. The Hallmark TNF-α signaling via NF-kB gene set was obtained from MSigDB and its score for each cell was calculated using AddModuleScore function in Seurat, which computes the average expression of genes in the gene set after subtracting the expression of matched control genes. The scores for each cell in both healthy and degenerated groups were mapped and visualized in UMAP plots. To identify genes contributing to the TNF-α signaling via NF-kB module score, the correlation between the expression of each gene in the gene set and the module score was calculated using Spearman correlation analysis, and the top correlated genes were visualized in a heatmap plot. Furthermore, whether the correlation of these genes and the module score was significantly different between healthy and degenerated samples was evaluated and adjusted *p* values were displayed in the same heatmap plot.

### 2.9. Quantitative Real-Time PCR Assays

Cultured human AF cells were lysed in TRIzol reagent (Thermofisher Scientific, #15596018), and total RNA was isolated according to the manufacturer’s instructions. The RNA concentration and purity were examined using a NanoDrop-1000 spectrophotometer (Thermofisher Scientific, #ND-2000). For cDNA synthesis, 1 mg of RNA was reverse-transcribed using the Verso cDNA synthesis kit (Thermofisher Scientific, #AB-1453B). The expression of target genes was examined by real-time qPCR using PowerUp SYBR Green Master Mix (Applied Biosystems, Waltham, MA, USA, #A25742). *ACTB* was used as the reference gene for normalization. Relative gene expression levels were calculated using 2^−ΔΔCT^ method. The primer sequences were listed in Table 2. Technical triplicates for each sample were performed. 

### 2.10. Statistics

To analyze the effects of the treatment on the expression of inflammatory and neurogenic genes in TNF-α stimulated cells, one-way analysis of variance (ANOVA) testing was performed, followed by Dunnett post hoc testing for multiple comparisons. Student’s *t*-tests were utilized to analyze the impact of PDGF-BB treatment on the expression of inflammatory and neurogenic genes at the basal levels in AF cells. Data are presented as mean ± standard deviations (SD). The statistical analyses were performed using GraphPad Prism 10. A *p*-value < 0.05 was considered statistically significant.

## 3. Results

### 3.1. Transcriptomic Responses to PDGF-BB Treatment in Human AF Cells

**Figure 1:**
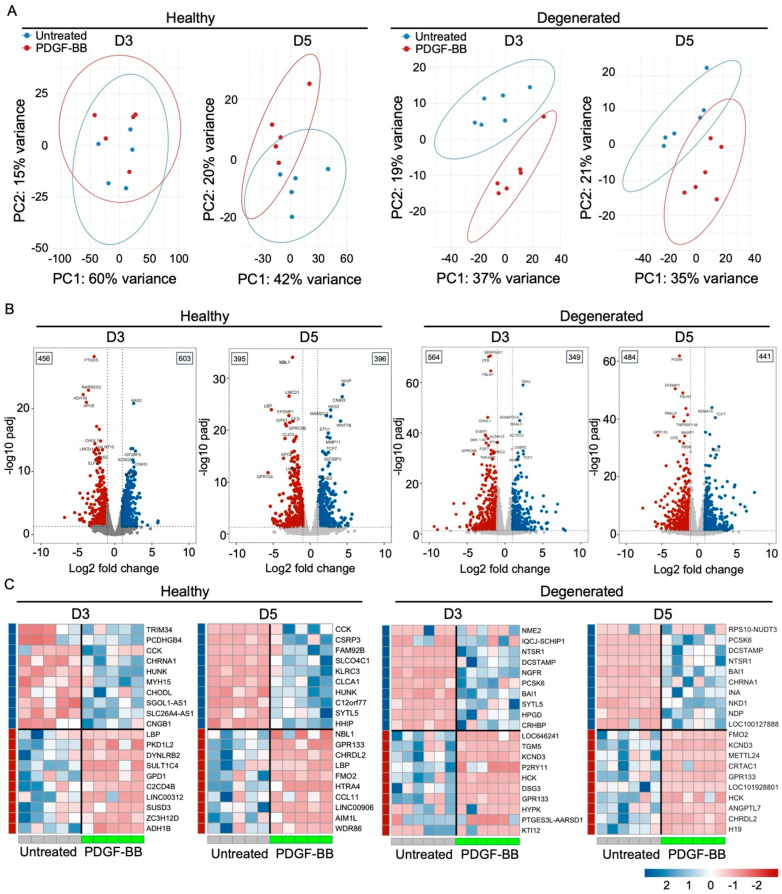
Transcriptomic responses to PDGF-BB treatment in human AF cells. (**A**) PCA plots showing the clustering of untreated and PDGF-BB treated samples at D3 and D5 in healthy and degenerated AF cells. (**B**) Volcano plots visualizing the magnitude and statistical significance of gene expression. The number of downregulated and upregulated differentially expressed genes (DEGs) in each group was shown in the box. Red indicates downregulated genes and blue indicates upregulated genes. Vertical and horizontal dashed lines indicate the threshold for log_2_ fold change (fold change = ±2) and −log_10_ padj (adjusted *p* value = 0.05) respectively. (**C**) Heatmaps showing the top 10 upregulated and downregulated DEGs (based on fold change) in each group. Each column represents the untreated and PDGF-BB treated replicates. Red indicates downregulated genes and blue indicates upregulated genes.

The global transcriptional impact of PDGF-BB on human AF cells was assessed using bulk RNA-seq. In healthy AF samples, PCA showed that untreated and PDGF-BB treated groups clustered together at day 3 (D3) but formed distinct clusters at day 5 (D5) (Figure 1A). In contrast, degenerated AF samples displayed distinct clustering between untreated and PDGF-BB groups at both timepoints. Differential expression analysis revealed that PDGF-BB treatment downregulated 456 genes and upregulated 603 genes at D3 in healthy AF, but fewer DEGs were detected at D5 (Figure 1B), suggesting that PDGF responses became stable over time. Several top DEGs (based on FDR) were consistently expressed across both timepoints in healthy AF cells, including Hyaluronan synthase 2 (*HAS2*), insulin-like growth factor 2 mRNA binding protein 3 (*IGF2BP3*), and Apolipoprotein E (*APOE*). In degenerated AF cells, DEG numbers remained consistent across timepoints, with around 900 upregulated and downregulated genes per timepoint (Figure 1B). We further showed the top DEGs with the largest absolute logFC in each group using heatmaps and found that many genes were inflammatory and metabolic mediators (e.g., LPS-binding protein (*LBP*), C-C motif chemokine ligand 11 (*CCL11*), and Flavin-containing monooxygenase 2 (*FMO2*)), extracellular matrix regulators (e.g., Chordin-like 2 (*CHRDL2*) and proprotein convertase subtilisin/kexin type 6 (*PCSK6*)), and neuronal and angiogenic markers (e.g., cholecystokinin (*CCK*) and potassium voltage-gated channel subfamily D member 3 (*KCND3*)) (Figure 1C).

### 3.2. Functional Clustering of PDGF-BB Regulated Genes in Human AF Cells

**Figure 2:**
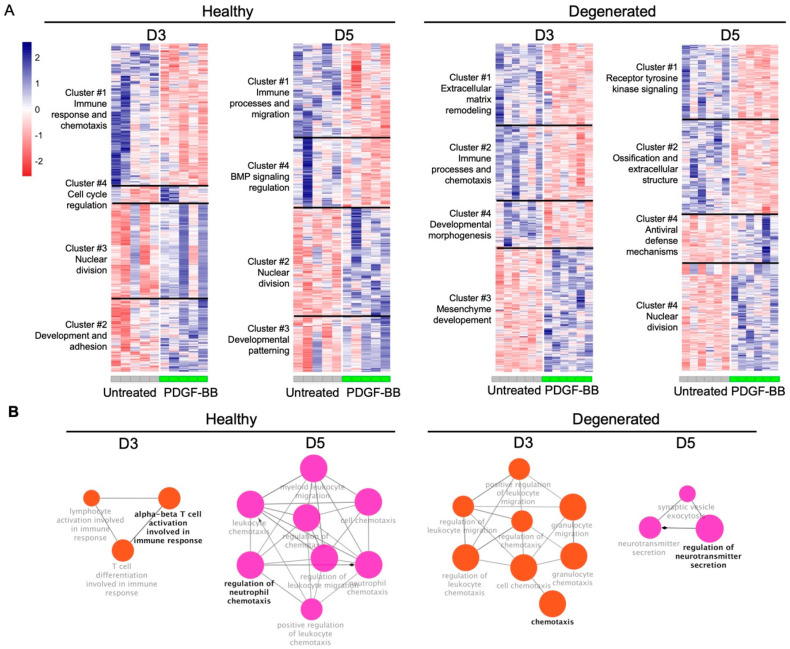
Functional clustering of PDGF-BB-regulated genes in human AF cells. (**A**) DEGs of each group were clustered according to their expression pattern into four clusters. Functional annotation of each cluster was performed using pathway enrichment analysis. Clusters were labeled based on the top five pathways. (**B**) Pathway network visualization of enriched gene ontology (GO) terms from all DEGs (without gene clustering) in each group. The network highlighted the major interconnected pathways. Black labels indicate the representative terms in each cluster, while gray labels indicate other interconnected GO terms. Arrows indicate direct relationships between two GO terms.

To determine the biological processes regulated by PDGF-BB, we performed functional enrichment analysis of the DEGs for each group. DEGs were grouped into four major clusters based on similarities in their expression patterns between untreated and PDGF-BB treated groups within each sample. Functional enrichment was performed for each cluster, which was annotated based on the most affected pathways (top five terms for each cluster were listed in Supplementary Figure S1). Therefore, the name of the clusters reflected the pathways that were most affected by the treatment, rather than all enriched biological processes. In healthy AF cells at D3, upregulated DEGs were enriched in pathways related to cell cycle progression and developmental growth while downregulated genes were associated with immune response and chemotaxis (Figure 2A). By D5, downregulation of BMP signaling also emerged among the most affected pathways (Figure 2A). In degenerated AF cells, the downregulated DEGs at D3 were involved in extracellular matrix organization, immune processes, and developmental morphogenesis while upregulated genes were primarily enriched in organ development (Figure 2A). At D5, downregulated genes were involved in signal transduction and ECM structure while upregulated genes were related to viral response and nuclear division (Figure 2A). Analysis of all DEGs as a whole without clustering confirmed that genes associated with immune response and neutrophil chemotaxis were consistently suppressed in healthy AF cells by PDGF-BB treatment at D3 and D5 (Figure 2B). In degenerated AF cells, the treatment inhibited chemotaxis-related genes at D3 and neurotransmitter-secretion-related genes by D5 (Figure 2B). As expected, upregulated DEGs were primarily involved in mitogenesis and organ morphogenesis in both healthy and degenerated AF cells (Supplementary Figure S2).

### 3.3. Consistent Gene Expression Changes Revealed Core Pathways Modulated by PDGF-BB

**Figure 3:**
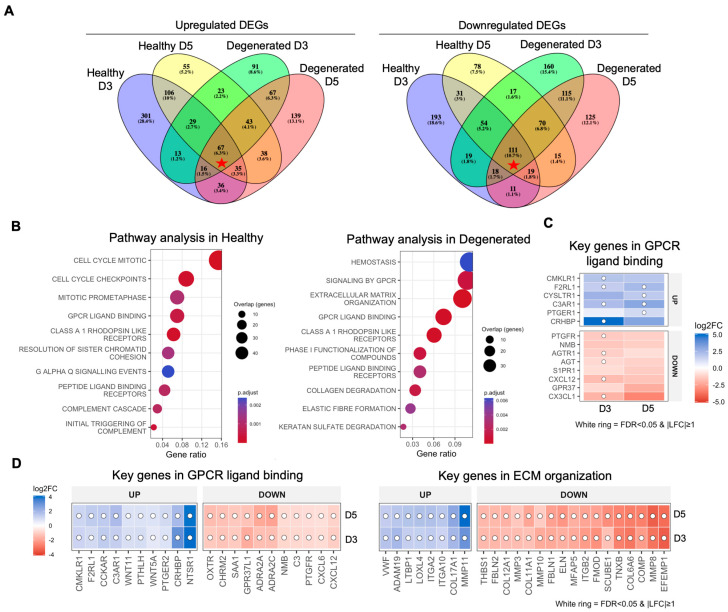
Consistent gene expression changes revealed core pathways modulated by PDGF-BB. (**A**) Venn diagrams showing the number of upregulated and downregulated DEGs at D3 and D5 in each condition. Red stars highlight the number of common DEGs regulated by PDGF-BB treatment in both healthy and degenerated AF cells at both timepoints. (**B**) Pathway enrichment of shared DEGs (both upregulated and downregulated) between D3 and D5 in each condition. Top 10 significant pathways are displayed in healthy or degenerated AF cells. The size of the bubble reflects the number of DEGs in the pathway. The color gradient indicates the adjusted *p* value. (**C**,**D**) Heatmaps visualizing the key genes of G protein-coupled receptors (GPCRs) signaling in healthy AF cells (**C**) at D3 and D5, and key genes of GPCR ligand binding and ECM organization in degenerated AF cells (**D**). The gene with a white ring indicates its adjusted *p* value ≤ 0.05 and |fold change (FC)| ≥ 2. The color scale represents the log_2_FC value of DEGs, with blue indicating increased DEGs and red indicating decreased DEGs.

Since transient gene expression changes may be lost with time and do not reflect the sustained biological effects of PDGF-BB, we next focused on the DEGs consistently expressed between D3 and D5. Venn diagrams (Figure 3A) provided a clear visualization of shared DEGs between timepoints and cell states. In healthy AF cells, 237 upregulated and 215 downregulated genes were shared between D3 and D5, while in degenerated AF cells, 193 upregulated and 314 downregulated genes overlapped across timepoints. We performed pathway enrichment analysis of shared DEGs (both upregulated and downregulated genes) and found that in healthy AF cells, PDGF-BB treatment consistently affected pathways related to cell cycle progression and complement cascade (Figure 3B). In degenerated AF cells, the shared DEGs were enriched in pathways linked to extracellular matrix organization, collagen degradation, keratan sulfate degradation, and elastic fiber formation (Figure 3B). Interestingly, both healthy and degenerated AF cells showed enrichment in GPCR-mediated signaling, including GPCR ligand binding, class A1 rhodopsin-like receptors, and peptide ligand-binding receptors, which were critical for chemokine, immune, and neuropeptide pathways. Heatmap confirmed the widespread downregulation of GPCR ligands and receptors (Figure 3). In healthy AF cells (Figure 3C), we observed the downregulation in ligands (e.g., C-X-C motif chemokine ligand (CXCL12), C-X3-C motif chemokine ligand 1 (CX3CL1), endothelin 1 (EDN1), neuromedin B (NMB), and angiotensinogen (AGT)) and receptors (e.g., AGT receptor 1 (AGTR1), G protein-coupled receptor 37 (GPR37), and Sphingosune-1-phosphate receptor 1 (S1PR1)). In degenerated AF cells (Figure 3D), the expression of additional receptors was suppressed, including oxytocin receptor (OXTR), cholinergic receptor muscarinic 2 (CHRM2), G protein-coupled receptor 37-like 1 (GPR37L1), adrenergic receptor alpha 2A/2C (ADRA2A/ADRA2C), and prostaglandin F receptor (PTGFR). In addition, complement component (C3) was downregulated whereas its receptor C3AR1 was upregulated in degenerated AF cells, suggesting that PDGF-BB treatment may regulate the expression levels of genes associated with complement pathway activity in degenerated AF cells. PDGF treatment was also correlated with changes in genes related to ECM organization pathway, with downregulation of matrix metalloproteinases (MMP3, 8, and 10) and ECM structural and regulatory genes, including thrombospondin-1 (THBS1), cartilage oligomeric matrix protein (COMP), and collagens (COL6A6, COL11A1, and COL12A1). Conversely, adhesion-related genes such as integrin subunit alpha 2 (ITGA2), ITGA10, and COL17A1 were upregulated in degenerated AF cells by PDGF-BB treatment. Together, these findings suggest that PDGF-BB consistently influenced the transcriptional changes in complement and GPCR-mediated immune and neuro signaling in both healthy and degenerated AF cells, but also exerted cell state-specific effects, with a stronger impact on cell cycle genes in healthy cells and a shift on ECM structural remodeling genes in degenerated cells.

### 3.4. Cell-State Specific Regulation of PDGF-BB in AF Cells

**Figure 4:**
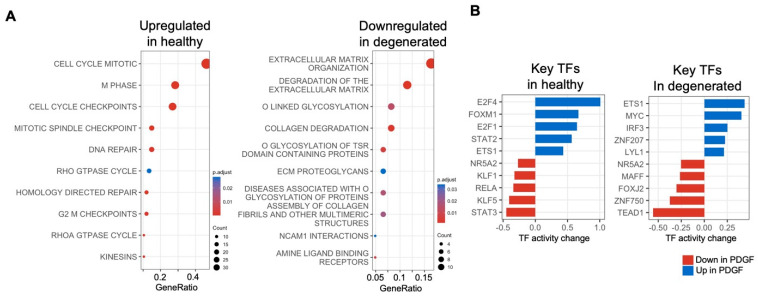
Cell-state specific regulation of PDGF-BB in AF cells. Pathway enrichment of cell-state unique DEGs (only present in either healthy or degenerated AF cells) in healthy (**A**) and degenerated (**B**) AF cells. In healthy cells, significant pathway enrichment was only detected in upregulated DEGs while in degenerated cells, enrichment was only observed in downregulated DEGs. The color gradient represents the adjusted *p* value. The size of the bubble reflects the number of DEGs in the pathway.

To further validate the differential effects of PDGF-BB between healthy and degenerated AF cells, we identified the DEGs that are uniquely regulated in each cell state and performed pathway enrichment on these gene sets. As shown in Figure 3A, 106 upregulated and 31 downregulated DEGs were uniquely regulated in healthy AF cells while 67 upregulated and 115 downregulated DEGs were uniquely regulated in degenerated AF cells (as indicated by red stars in Figure 3A). Functional enrichment analysis showed that the significant pathways were detected only among the upregulated DEGs in healthy AF cells and in degenerated cells significant pathways were detected only among downregulated DEGs. Consistent with earlier findings, cell cycle related pathways were enriched in healthy AF cells (Figure 4A) while in degenerated cells, suppression of ECM remodeling pathways were detected, including degradation of matrix and collagen degradation, neural cell adhesion molecule 1 (NCAM1) interaction, and amine ligand binding receptors (Figure 4B).

### 3.5. Conserved Responses to PDGF-BB Across AF Cell States

**Figure 5:**
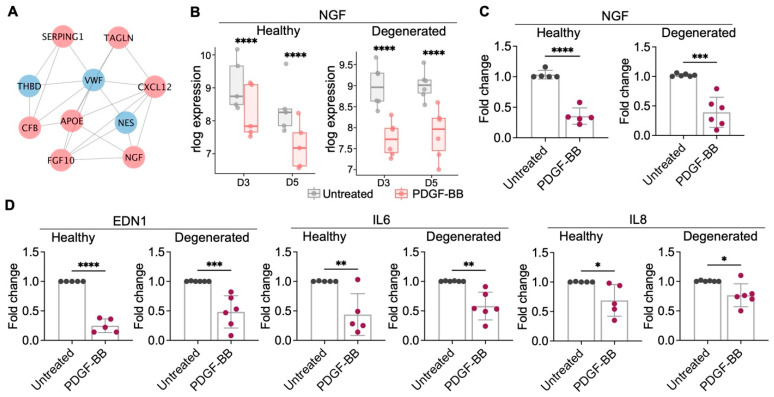
Conserved responses to PDGF-BB across AF cell states. (**A**) Protein–protein interaction (PPI) network built from common DEGs that were regulated by PDGF-BB in both healthy and degenerated AF cells. Top 10 hub genes are displayed. Upregulated DEGs are labeled blue and downregulated are labeled red. (**B**) The normalized expression of nerve growth factor (*NGF*) from bulk-seq data at D3 and D5 in each condition. **** *p* < 0.0001. (**C**) The gene expression levels of *NGF* in untreated and PDGF-BB treated groups in each condition. The cells were treated by rhPDGF-BB (20 ng/mL) for 5 days. Healthy: *n* = 5. Degenerated: *n* = 6. Student’s *t* test was used to analyze the effects of PDGF-BB in each condition. Data are presented as mean with SD. *** *p* < 0.001. **** *p* < 0.0001. (**D**) The gene expression of endothelin 1 (*EDN1*), interleukin (*IL*) 6, and *IL8* in healthy and degenerated AF cells after rhPDGF-BB treatment for 5 days. Healthy: *n* = 5. Degenerated: *n* = 6. Student *t* test was used to analyze the effects of PDGF-BB in each condition. Data are presented as mean with SD. * *p* < 0.05. ** *p* < 0.01. *** *p* < 0.001. **** *p* < 0.0001.

In addition to state-specific responses, we evaluated the fundamental and conserved effects of PDGF-BB that were independent of cell state. Figure 3A showed that 67 upregulated and 111 downregulated DEGs were shared between healthy and degenerated AF cells in response to the treatment. The PPI network was constructed from these shared DEGs, which revealed the top 10 highly interconnected genes (Figure 5A). These hub genes involved in endothelial and vascular regulation such as von Willebrand factor (*VWF*) and thrombomodulin (*THBD*), as well as inflammatory and neurogenic mediators such as *CXCL12*, apolipoprotin E (*APOE*), and nerve growth factor (*NGF*), suggesting the conserved regulation of vascular, immune, and neurotrophic pathways by PDGF-BB treatment in both healthy and degenerated AF cells. RNA-seq expression profiling further showed that *NGF* was consistently downregulated by PDGF-BB treatment at both D3 and D5 in each cell state (Figure 5B). This downregulation was further validated by gene expression analysis at D5. In addition, *EDN1*, *IL6*, and *IL8* gene expression was also inhibited by PDGF-BB, as revealed by RNA-seq (Supplementary Figure S3) and qPCR analyses (Figure 5C), confirming that PDGF-BB broadly attenuated pro-inflammatory and neurogenic mediators in both healthy and degenerated human AF cells.

### 3.6. scRNA-Seq Profiling Revealed the Association Between NGF and TNF-α/NF-kB Signaling in Human AF

**Figure 6:**
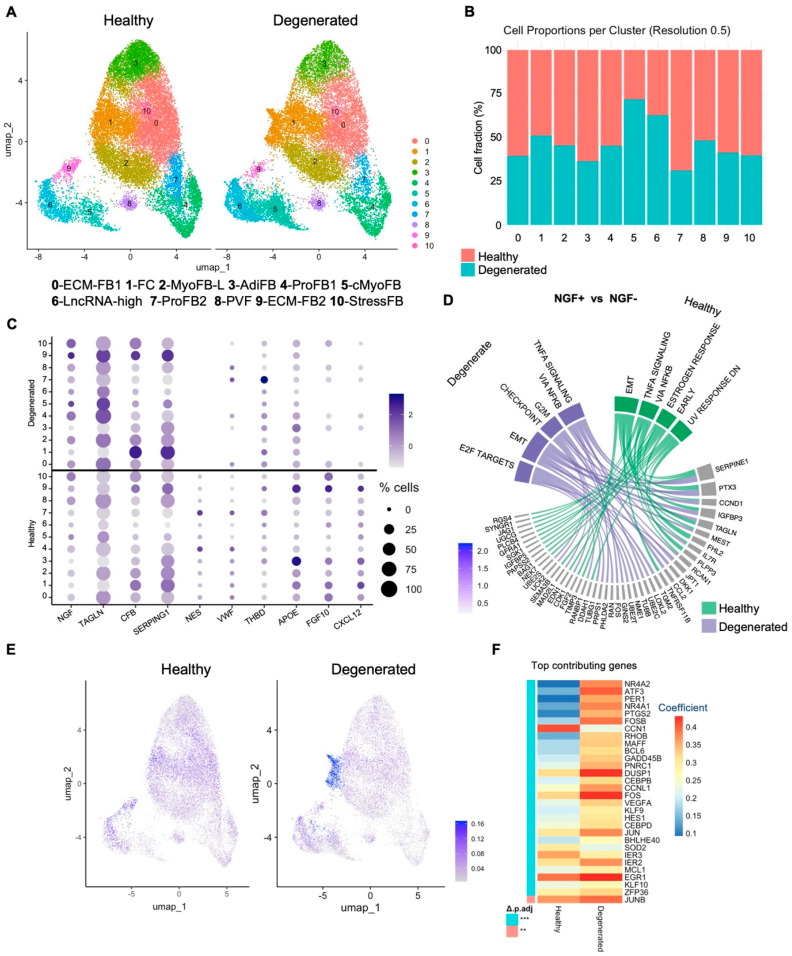
scRNA-seq profiling revealed the association between NGF and TNF-α/NF-kB signaling in human AF. (**A**) UMAP plots (resolution = 0.5) revealed 11 clusters in both healthy and degenerated AF cells. Multiple fibroblast subtypes were identified. Each color represents a cluster. ECM-FB1: extracellular matrix rich fibroblast 1. FC: fibrochondrocyte. MyoFB-L: myofibroblast-like fibroblast. AdiFB: adipogenic fibroblast. ProFB1: proliferative fibroblast 1. cMyofb: contractile myofibroblast 1. LncRNA-high: Long non-coding RNA high fibroblast. ProFB2: proliferative fibroblast 2. PVF: perivascular fibroblast. ECM-FB2: extracellular matrix rich fibroblast 2. StressFB: stress-response fibroblast. (**B**) Stacked bar plot of cluster proportions by condition. Healthy cells showed a lower proportion of proliferative fibroblasts (cluster 7) and fibrocartilage cells (cluster 3). Contractile myofibroblasts (cluster 5) and long non-coding RNA high cells (cluster 6) were increased in degenerated AF. (**C**) Dot plot mapping top 10 PPI hub genes (from bulk RNA-seq) onto single-cell clusters. The black line indicates the separation between healthy and degenerated samples. (**D**) Pathway enrichment analysis of *NGF* co-expressed genes in each condition. Both healthy and degenerated AF cells showed enrichment for epithelial–mesenchymal transition (EMT) and TNF-α/NF-kB pathways in *NGF* positive cells. Degenerative cells additionally showed E2F targets/G2M checkpoint higher proliferative potential. (**E**) Feature plots showing the distribution of TNF-α/NF-kB signaling in healthy and degenerated AF cells. (**F**) Top contributing genes of TNF-α/NF-kB signaling activity in healthy and degenerated AF cells. Color gradient represents the coefficient factor of each gene in the signaling. ** *p* < 0.01. *** *p* < 0.001.

To assess whether the pathways regulated by PDGF-BB were present in native human AF cells, we analyzed a publicly available scRNA-seq dataset comprising three healthy and six degenerated (Thompson grade 3) human AF samples. As shown by Figure 6A, 11 clusters were identified, with multiple fibroblast subtypes. Notably, degenerated samples showed a higher cell proportion in cluster 5 (contractile myofibroblasts) and 6 (long non-coding RNA high cells) (Figure 6B), indicating expansion of contractile and stressed cell population with IVD degeneration. From bulk RNA-seq, the top 10 hub genes from PPI analysis were identified (Figure 5A). We mapped their expression across each cluster in both conditions in single-cell data and found that degenerated cells were enriched for *NGF*, *TAGLN*, *CFB*, and *SERPING1* (Figure 6C), consistent with their suppression by PDGF-BB treatment (Figure 5A). By contrast, *NES* and *VWF*, which were induced by PDGF-BB treatment, were slightly enriched in healthy AF cells. *APOE*, *FGF10*, and *CXCL12* were lower in degenerated cells and were further inhibited by PDGF-BB treatment.

NGF, a neurotrophic factor, promotes nerve ingrowth and pain sensation. To explore the *NGF*-associated signaling in AF cells, we analyzed *NGF*-positive and *NGF*-negative population in each condition. In healthy AF cells, *NGF* co-expressed genes were enriched for EMT, TNF-α signaling via NF-kB, early estrogen response, and UV response (down) (Figure 6D). In degenerated AF cells, in addition to EMT and TNF-α signaling via NFkB, cell cycle-related pathways were also enriched in *NGF*-positive population, suggesting greater proliferative potential of *NGF* positive cells in degenerated AF (Figure 6D). Key genes such as *SERPINE1*, *PTX3*, *CCND1*, and *IGFBP3* were involved in these pathways. To examine which AF cells populations showed strong TNF-α/NF-kB activity, the expression of genes from the Hallmark TNF-α signaling via NFkB were scored for each cell and the resulting scores were mapped in UMAP plots. We found that healthy cells showed relatively low activity of this pathway while degenerated AF cells displayed a compact high-density niche in a subset of cluster 1 (Figure 6E). The top contributing gene to this signature in healthy cells is cellular communication network factor 1 (*CCN1*), while early growth response 1 (*EGR1*), Fos proto oncogene (*FOS*), and dual-specificity phosphate 1 (*DUSP1*) exhibited stronger coefficients in degenerated cells (Figure 6F). Taken together, these results indicate that *NGF*-positive cells were engaged in TNF/NF-kB and EMT pathways, with additional proliferative signaling in degenerated AF.

### 3.7. PDGF-BB Mitigated Inflammatory Responses Under TNF-α Stimulation

**Figure 7:**
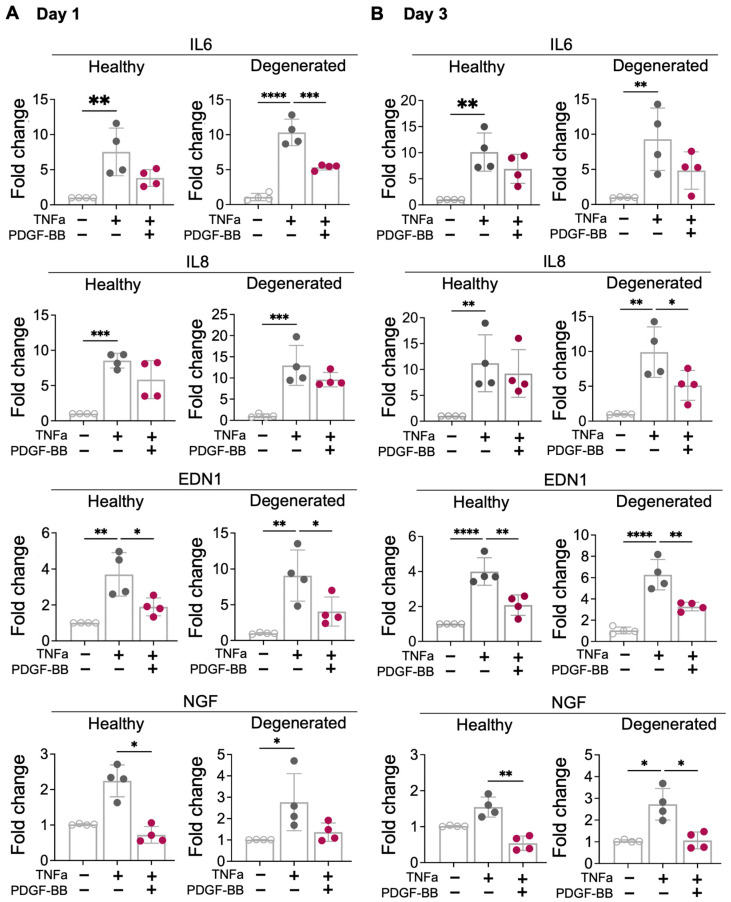
PDGF-BB mitigated inflammatory responses under TNF-α stimulation. The gene expression of proinflammatory and neurotrophic mediators *IL6*, *IL8*, *EDN1*, and *NGF* in healthy and degenerated AF cells when stimulated with rhTNF-α (20 ng/mL) in the presence or absence of rhPDGF-BB (20 ng/mL) for 1 (**A**) and 3 (**B**) days. Healthy and degenerated samples: *n* = 4. One-way ANOVA with Dunnett post hoc testing was performed. Data are presented as mean with SD. * *p* < 0.05. ** *p* < 0.01. *** *p* < 0.001. **** *p* < 0.0001.

We next examined whether TNF-α could stimulate the expression of *NGF* and whether PDGF could attenuate this response in AF cells, mimicking the inflammatory environment of degenerated IVDs. To test this, healthy and degenerated AF cells were stimulated with rhTNF-α (20 ng/mL), in the presence or absence of rhPDGF-BB (20 ng/mL) for 1 and 3 days. As expected, TNF-α significantly induced the expression of *IL6* and *IL8* in both healthy and degenerated AF cells at day 1 (Figure 7A) and day 3 (Figure 7B). PDGF-BB reduced their expression in degenerated AF cells, while this effect did not reach statistical significance in healthy AF cells. *EDN1* was consistently increased by TNF-α in both cell states at both timepoints, which was inhibited by PDGF-BB treatment (Figure 7A,B). In addition, TNF-α induced *NGF* expression in both healthy and degenerated AF cells at D3 while PDGF-BB reduced its expression in healthy AF cells at D1 and degenerated AF cells at both D1 and D3 (Figure 7A,B). Collectively, these findings suggest that PDGF-BB can mitigate the expression of proinflammatory and neurogenic mediators in AF cells not only at baseline but also in the presence of TNF-α stimulation.

## 4. Discussion

In this study, we explored the time- and state-dependent transcriptomic responses of human AF cells to PDGF-BB treatment and identified PDGF-BB as a potential suppressor of inflammation-neurogenic pathways at the transcriptional level in the IVD. Our findings indicate that PDGF-BB treatment exerted both shared and cell-state-specific effects. In both healthy and degenerated AF cells, PDGF-BB consistently upregulated cell cycle-related genes while downregulating the genes associated with inflammatory, neurogenic, and immune signaling, including key mediators such as *NGF*. These shared transcriptomic responses suggest that PDGF-BB may have a conserved regulatory effect across different AF cell states. However, cell-state-specific responses to PDGF-BB were also observed. In healthy AF cells, PDGF-BB increased the genes related to cell cycle and developmental growth. In degenerated AF cells, PDGF-BB reduced the expression of genes involved in extracellular matrix degradation in degenerated AF cells, suggesting a transcriptional shift toward a more reparative and structure stabilizing state. To relate these PDGF-BB-induced transcriptomic changes to disc degeneration, we further analyzed published human AF single-cell RNA-seq datasets and found that inflammatory and neurogenic mediators including *NGF* were enriched in degenerated AF cell populations. Furthermore, *NGF*-positive cells showed strong TNF-α/NF-kB pathway activity. To examine the relation between NGF and TNF-α, we stimulated human AF cells with TNF-α and found that TNF-α induced *NGF* expression, while PDGF-BB suppressed this effect. Together, these findings indicate that PDGF-BB modulates transcriptomic activity in multiple pathological pathways, including inflammation, neurogenic signaling, and matrix remodeling, that are hallmarks of disc degeneration.

Degenerated AF cells reside within a chronic inflammatory microenvironment. It is well-known that the expression levels of pro-inflammatory cytokines such as IL-1α and TNF are increased in degenerated disc cells [24,25]. The avascular nature of the disc makes the inflammatory cytokines produced by local disc cells not being sufficiently cleared from the tissue, contributing to persistent inflammation [26]. Consistently, as revealed by our single-cell RNA seq analysis, we found an expanded subset of AF cells in degenerated samples expressing a remarkably high level of genes involved in the TNF-α/NF-kB pathway in degenerated AF cells. Top contributing genes of this pathway include transcription factors *EGR1* and *FOS*, both of which trigger inflammation-mediated disease in other tissues such as the lung and skin [27,28]. In rat tail IVD puncture models, selective inhibition of c-Fos/Activator protein-1 (*AP-1*) by T-5224 or *EGR1* knockdown alleviated disc degeneration, supporting that targeting inflammatory pathways holds great potential for treating disc degeneration [29,30]. When human healthy and degenerated AF cells were treated with PDGF-BB, the most prominent effects on both groups were a reduction in the expression of genes involved in inflammation, immune response and chemotaxis, which are the most pronounced effects observed. Notably, degenerated cells showed a stronger transcriptional response to PDGF-BB than healthy cells, probably reflecting increased PDGF receptor activity in these cells. Furthermore, PDGF-BB also attenuated the increased expression of pro-inflammatory mediators by TNF-α, suggesting a reduction in both basal and TNF-α induced inflammation in AF cells. However, differential responses to TNF-α stimulation were observed between healthy and degenerated AF cells, with more consistent upregulation in the expression of inflammatory cytokines and *NGF* in degenerated AF cells. One possible explanation is that degenerated cells have higher basal inflammatory levels and altered cytokine-responsive transcriptional profile, making them more susceptible to the external stimuli. 

With degeneration, damaged AF facilitates the infiltration of immune cells, macrophages, and neovascularization [31,32]. Inflammatory cytokines released by the resident cells or infiltrating cells promote matrix degradation and upregulate neurotrophins such as *NGF* [12,26]. These changes induce the ingrowth of nociceptive nerve fibers, accompanied by blood vessels [33,34]. A key finding of this study is the consistent downregulation of *NGF* by PDGF-BB treatment in both healthy and degenerated AF cells, which is also a central hub gene identified by PPI network analysis. The majority of DRG neurons innervating into the disc are *NGF*-sensitive, and the expression level of *NGF* is positively correlated with innervation density in IVDs [35,36,37]. In addition, previous studies have shown that pro-inflammatory cytokines such as IL-1β and TNF-α stimulate the expression of *NGF* in human IVD cells [22,38]. Our single-cell RNA seq analysis revealed that *NGF*-positive AF subpopulations were enriched for TNF-α/NF-kB and EMT pathways, consistent with a pro-inflammatory and stress-responsive state. PDGF-BB treatment suppressed the expression of *NGF* both at baseline and under TNF-α stimulation. Given the critical role of *NGF* in promoting nociceptive sensitization and nerve ingrowth, its observed reduction in *NGF* expression may represent a potential transcriptomic mechanism through which PDGF-BB modulates the transcriptomic activity of pain-related pathways in disc degeneration. Future work will be required to determine whether these transcriptional effects of PDGF-BB could translate to reduced discogenic pain and enhanced tissue repair utilizing pre-clinical IVD degeneration models. 

In addition to its effects on inflammation-neurotrophic signaling, PDGF-BB regulated the transcriptomic shift in GPCR signaling in both healthy and degenerated AF cells, suggesting a potential unrecognized role of PDGF in modulating intercellular communication in the AF. GPCRs, the largest family of membrane receptors, allow cells to sense and respond to a variety of extracellular cues, including hormones, neurotransmitters, lipids, and chemokines, which makes them the most intensively studied drug targets [39,40]. We showed that PDGF-BB downregulated the mRNA levels of the GPCR ligands and receptors that are involved in inflammatory and neurogenic signaling, such as *CXCL6*, *CXCL12*, *NMB*, and *ADRA2A/C*, while upregulating mediators associated with matrix-remodeling and resolution of inflammation such as *CMKLR1*, *F2RL1* and *WNT11*. Notably, Kupka and colleagues characterized the expression levels and distribution of adrenergic receptors in human IVDs and found that *ADRA2A* was mainly localized in the AF tissue, and it was also expressed in cultured AF cells, confirming the existence of sympathetic neurotransmitter within the AF [41]. When stimulated with the neurotransmitter norepinephrine, AF cells showed increased ERK phosphorylation, suggesting that neurotransmitters can directly modulate intracellular signaling in the AF [41]. Consistent with this, Feng and colleagues demonstrated that human non-degenerated AF cells express mesenchymal and neuronal stem cell markers [42]. These cells are capable of neurogenic differentiation when cultured under neurogenic stimuli, with the neurite formation and neurofilament light chain expression [42]. Together, these results indicate that the AF cells possess neurogenic potential that may respond to sympathetic and inflammatory cues within the degenerating disc, while PDGF-BB treatment could suppress the transcriptional levels of *ADRA2A/C* and other neurogenic GPCRs in human AF cells.

PDGF-BB also modulated ECM gene expression, particularly in degenerated AF cells, suggesting its involvement in the structure and mechanobiology in the AF. Genes associated with matrix/collagen degradation and turnover, such as *MMP3/8/10*, *THBS1*, and *COMP* were consistently downregulated by PDGF-BB at both D3 and D5. *THBS1*, recently identified as a pathogenic signal in both NP and AF, is associated with fibrotic remodeling and angiogenesis while *COMP* accumulation correlates with matrix disorganization in degenerative IVDs [43]. The suppression of these genes together with the upregulation of genes such as *ITGA4*, *ITGA10*, and *COL17A1* that are involved in matrix adhesion and crosslinking suggest that PDGF-BB promotes a transcriptomic shift toward a more organized and structurally stabilizing state in AF tissues. 

While our study provides transcriptomic insights into the effects of PDGF-BB in AF cells, we must consider the limitations when interpreting these findings. The sample size in this study is relatively small with five donors for healthy tissues and six donors for degenerated tissues, which may limit the statistical power and not fully capture the inter-donor variability. Although PCA was used to assess the donor variability and potential batch patterns and showed distinct clustering between untreated and PDGF-BB-treated samples in degenerated AF, the findings may not be fully generalized to the overall patient population due to the heterogeneity of disc degeneration. Additionally, the age difference between the healthy and degenerated groups introduces a potential confounding factor when interpreting the responses to PDGF-BB treatment. It is possible that aged but non-degenerated AF cells, or young but degenerated AF cells may respond differently to PDGF-BB compared with aged and degenerated AF cells. Although age-matched healthy and degenerated AF tissues would be ideal for separating age-related and degeneration-related effects, access to such donor tissues is limited because they are either rare or difficult to obtain clinically. Our within-group comparison between untreated and treated AF cells provides insight into how PDGF-BB modulates transcriptomic responses in patient-derived AF cells. Although Bulk RNA-seq and qPCR analyses suggest that PDGF-BB reduced the expression of genes associated with inflammatory and neurogenic signaling, it does not guarantee similar changes at the protein levels. Furthermore, PDGF-BB is known to bind its receptors, but we did not observe any significance changes in the expression of PDGF receptors based on the transcriptomic data. It is possible that receptor activity is regulated at the level of post-translational level, rather than at the transcriptional level. Future mechanistic and functional assays are also required to validate the transcriptomic findings and determine the impact of the observed gene expression changes and whether these responses are mediated through PDGF receptors. Given that the in vitro monolayer culture system may not fully represent the complex 3D of the native IVDs, the observed transcriptional responses may differ in vivo. In addition, long-term treatment functional validation is necessary to confirm the sustained protective roles of PDGF-BB in the AF.

## 5. Conclusions

Our results indicate that PDGF modulates the transcriptomic profile by suppressing inflammation and neurogenic signaling while simultaneously upregulating genes associated with ECM remodeling in the AF. These findings provide a foundation for further investigation into the role of PDGF-BB in structural degeneration and nociceptive signaling in degenerated IVDs.

## Figures and Tables

**Table 1:** Donor information.

Grade	Age	Sex
4	67	M
5	61	M
5	61	M
4 or 5	65	F
4 or 5	81	F
4	53	F
1	19	M
1	21	M
1	20	M
1	25	F
1	27	F

**Table 2:** Sequence of primers.

Gene	GEO Accession Number	Primer Sequence	Amplicon Size
*IL6*	NM_000600.5	Forward: 5′-CCGGGAACGAAAGAGAAGCT-3′ Reverse: 5′-GCGCTTGTGGAGAAGGAGTT-3′	68
*IL8*	NM_000584.4	Forward: 5′-CTTTCCACCCCAAATTTATCAAAG-3′ Reverse: 5′-CAGACAGAGCTCTCTTCCATCAGA-3′	107
*EDN1*	NM_001955.5	Forward: 5′-CAGCAGTCTTAGGCGCTGAG-3′ Reverse: 5′-ACTCTTTATCCATCAGGGACGAG-3′	126
*NGF*	NM_002506.3	Forward: 5′-CAGCAGGAAGGCTGTGAGAA-3′ Reverse: 5′-TACAGGTTGAGGTAGGGAGGG-3′	96
*ACTB*	NM_001101.5	Forward: 5′-CTCTTCCAGCCTTCCTTCCT-3′ Reverse: 5′-AGCACTGTGTTGGCGTACAG-3′	116

Note: *IL6*: interleukin 6. *IL8*: interleukin 8. *EDN1*: endothelin 1. *NGF*: nerve growth factor. *ACTB*: β-actin.

## Data Availability

The resulting sequences were uploaded into the NCBI Sequence Read Archive (SRA) database (PRJNA1354819). The code used during the current study is available from the corresponding author on reasonable request.

## Supplementary Materials

The following supporting information can be downloaded at https://www.mdpi.com/article/10.3390/cells15111007/s1, Figure S1: Pathway enrichment analysis for the DEGs by PDGF-BB treatment in human AF cells. DEGs, clustered based on the expression dynamics, were used as an input for biological pathway enrichment. Top five pathways in each cluster were displayed; Figure S2: Pathway enrichment network analysis for the upregulated DEGs by PDGF-BB treatment in human AF cells. Upregulated DEGs (without clustering) were used for pathway enrichment network analysis. The major network from each group was shown. Figure S3: PDGF-BB altered inflammatory response related gene expression in human AF cells. Heatmap showing the changes of genes involved in inflammatory response pathway at D3 and D5 following PDGF-BB treatment in each condition. Each column represents individual sample. Each row represents a gene. Blue indicates upregulated genes and red indicates downregulated genes.
