# Supplementary figures and images for: Transcriptomic Signature of PDGF-BB Control of Annulus Fibrosus Reveals Modulation of Inflammatory and Neurogenic Pathways
Source: Cells. 2026 May 30;15(11):1007. doi: 10.3390/cells15111007 (PMC13257205; doi:10.3390/cells15111007)

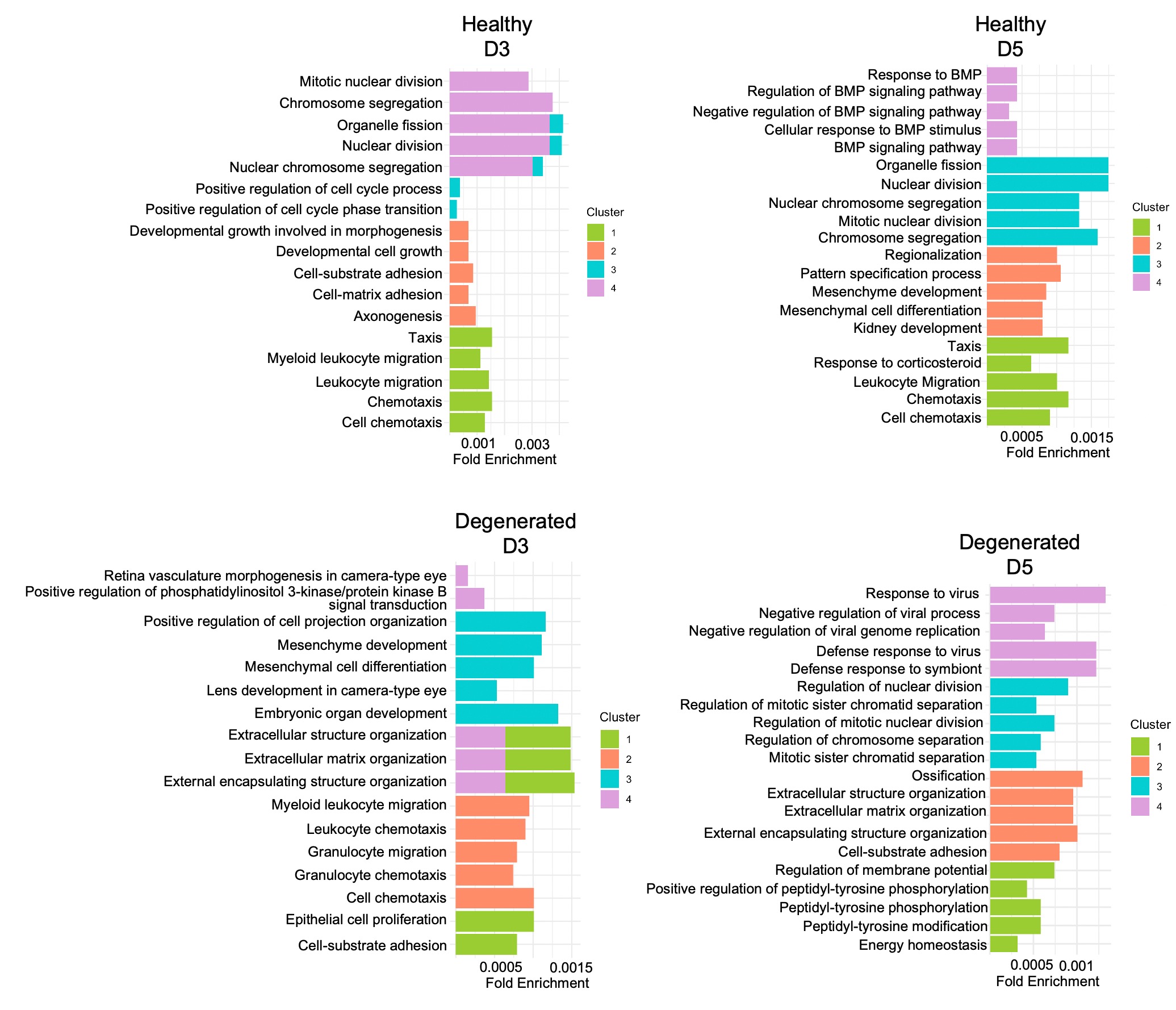

Supplement: Supplementary file 1 [file cells-15-01007-s001.zip › SF1.jpg]

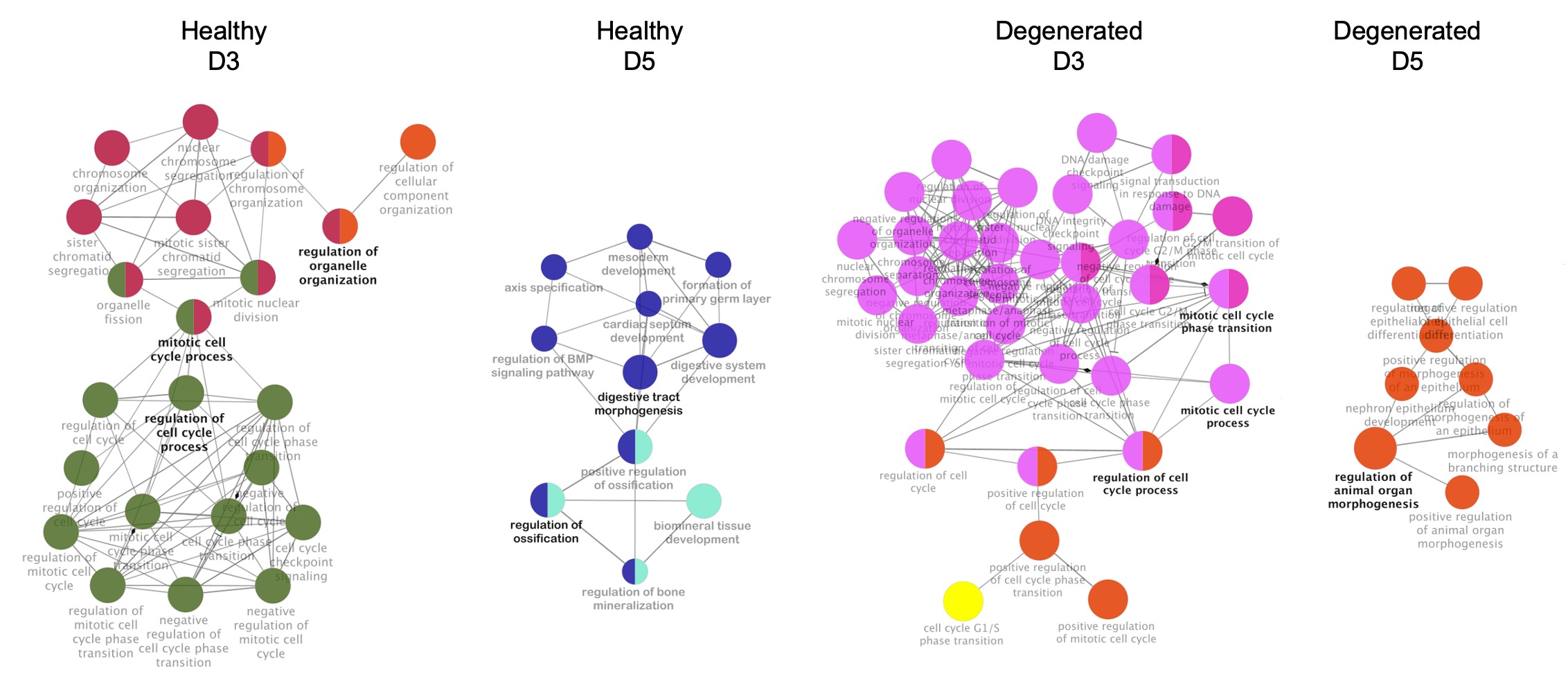

Supplement: Supplementary file 1 [file cells-15-01007-s001.zip › SF2.jpg]

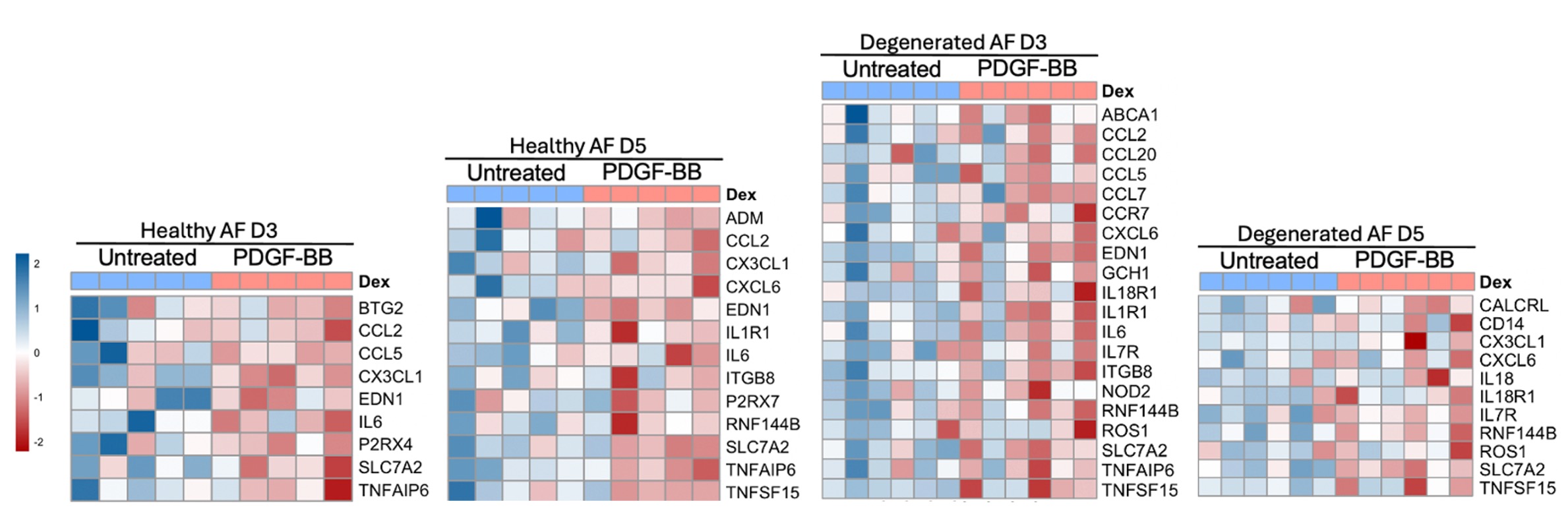

Supplement: Supplementary file 1 [file cells-15-01007-s001.zip › SF3.jpg]
